# Host- and Microbe-Dependent Dietary Lipid Metabolism in the Control of Allergy, Inflammation, and Immunity

**DOI:** 10.3389/fnut.2019.00036

**Published:** 2019-04-10

**Authors:** Azusa Saika, Takahiro Nagatake, Jun Kunisawa

**Affiliations:** ^1^Laboratory of Vaccine Materials, Center for Vaccine and Adjuvant Research, and Laboratory of Gut Environmental System, National Institutes of Biomedical Innovation, Health and Nutrition, Osaka, Japan; ^2^Graduate School of Pharmaceutical Sciences, Osaka University, Osaka, Japan; ^3^International Research and Development Center for Mucosal Vaccines, Institute of Medical Science, University of Tokyo, Tokyo, Japan; ^4^Graduate School of Medicine, Graduate School of Dentistry, Osaka University, Osaka, Japan; ^5^Graduate School of Medicine, Kobe University, Kobe, Japan

**Keywords:** lipid metabolites, dietary oil, intestinal immunity, inflammation, allergy, intestinal bacteria

## Abstract

The intestine is the largest immune organ in the body, provides the first line of defense against pathogens, and prevents excessive immune reactions to harmless or beneficial non-self-materials, such as food and intestinal bacteria. Allergic and inflammatory diseases in the intestine occur as a result of dysregulation of immunological homeostasis mediated by intestinal immunity. Several lines of evidence suggest that gut environmental factors, including nutrition and intestinal bacteria, play important roles in controlling host immune responses and maintaining homeostasis. Among nutritional factors, ω3 and ω6 essential polyunsaturated fatty acids (PUFAs) profoundly influence the host immune system. Recent advances in lipidomics technology have led to the identification of lipid mediators derived from ω3- and ω6-PUFAs. In particular, lipid metabolites from ω3-PUFAs (e.g., eicosapentaenoic acid and docosahexaenoic acid) have recently been shown to exert anti-allergic and anti-inflammatory responses; these metabolites include resolvins, protectins, and maresins. Furthermore, a new class of anti-allergic and anti-inflammatory lipid metabolites of 17,18-epoxyeicosatetraenoic acid has recently been identified in the control of allergic and inflammatory diseases in the gut and skin. Although these lipid metabolites were found to be endogenously generated in the host, accumulating evidence indicates that intestinal bacteria also participate in lipid metabolism and thus generate bioactive unique lipid mediators. In this review, we discuss the production machinery of lipid metabolites in the host and intestinal bacteria and the roles of these metabolites in the regulation of host immunity.

## Introduction

Lipid composition in organisms differs among species, in accordance with the expression levels of metabolic enzymes and dietary habits. Marine phytoplankton and seaweeds produce a large amount of the ω3-polyunsaturated fatty acids (PUFAs) eicosapentaenoic acid (EPA) and docosahexaenoic acid (DHA) (1). Although fish do not generate EPA and DHA per se, they accumulate EPA and DHA by eating phytoplankton (1). In plants, linseed and perilla contain large amounts of α-linolenic acid, a precursor of EPA and DHA. In contrast, soybean oil and sesame oil contain copious quantities of the ω6-PUFA linoleic acid. The difference in the fatty acid composition of plants depends on the expression levels and activities of metabolic enzymes such as Δ12-desaturase and Δ15-desaturase, which are involved in the generation of linoleic acid and α-linolenic acid, respectively (2, 3). Because mammals do not have either Δ12 or Δ15-desaturase, ω3- and ω6-PUFAs are categorized as essential fatty acids that must be obtained from the diet (3). Therefore, the balance of ω3 and ω6 lipids in the body largely depends on the quality of the dietary lipid consumed.

The beneficial effect of dietary ω3-PUFAs on human health was first reported in an epidemiological study in 1978 in which Greenland Eskimos, who consume high ω3-PUFA diets that include fish, were found to have a lower mortality from coronary heart disease than Danes and Americans, who eat much less ω3-PUFAs (4). Since then, accumulating evidence indicates that EPA and DHA have beneficial effects on the inhibition of various types of inflammatory and allergic diseases, including cardiovascular disease, Alzheimer's disease, rheumatoid arthritis, inflammatory bowel disease, atopic dermatitis, asthma, and food allergy (5–13). Recent developments in analytical technology, including liquid chromatography (LC) and mass spectrometry (MS), have enabled us to identify EPA- and DHA-derived pro-resolving lipid mediators (SPMs), including resolvins (Rvs), protectins (PDs), maresins (MaRs), and 17,18-epoxyeicosatetraenoic acid (17,18-EpETE) for inhibition of inflammatory and allergic diseases (7, 14).

Dietary lipids are metabolized not only by mammalian enzymes but also by bacterial enzymes. Microorganisms can generate unique lipid metabolites such as conjugated linoleic acids, hydroxy fatty acids, and oxo fatty acids. These bacteria-produced lipid metabolites show biological activity in the context of host health and diseases (15, 16). Here, we review our current understanding of ω3- and ω6-PUFA-derived lipid mediators in the control of inflammatory and allergic diseases.

## ω6 Fatty Acid Metabolites Have Opposing Roles in Pro-and Anti-Inflammation

**Figure 1:**
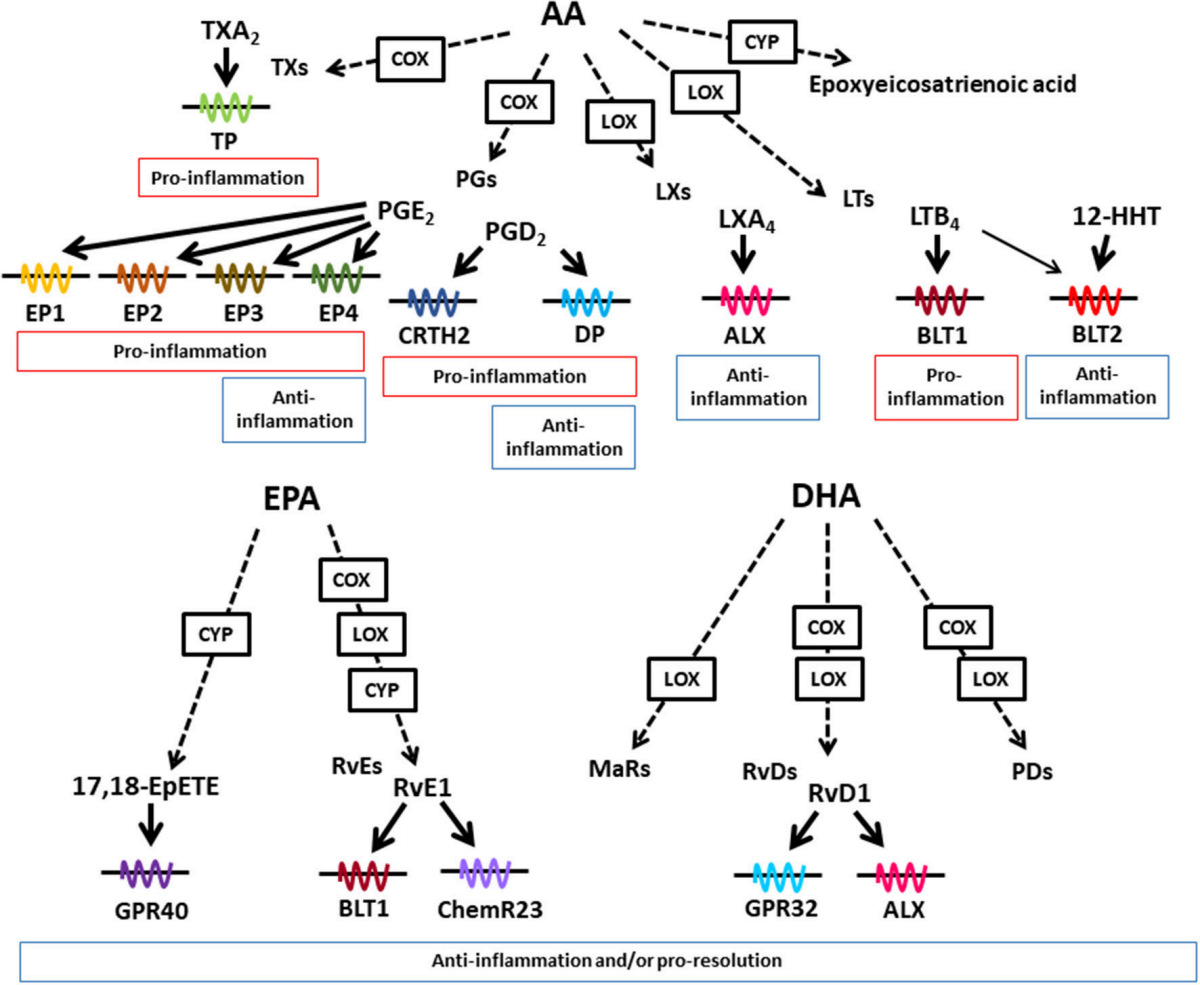
Lipid mediators derived from AA, EPA, and DHA. Various kinds of lipid mediators are produced from ω6- and ω3-PUFAs. AA, EPA, and DHA are converted to bioactive lipid mediators by the enzymatic activities of COX, LOX, and CYP. Lipid mediators exert their biological effects through binding to G-protein-coupled receptors. AA-derived lipid mediators have pro- and anti-inflammatory activities, whereas EPA- and DHA-derived lipid mediators exert anti-inflammatory or pro-resolution activities or both.

Dietary lipids are metabolized in the body to lipid mediators, which regulate host immune systems. Arachidonic acid (AA) is a metabolite of linoleic acid, and functions as a direct precursor of bioactive lipid mediators, which are known as eicosanoids. In addition to its biosynthesis in the body from linoleic acid, AA can be obtained from dietary sources, such as meat and eggs. AA is metabolized by cyclooxygenase (COX), lipoxygenase (LOX), and cytochrome P450 (CYP), and then converted into lipid mediators, including prostaglandins (PGs), leukotrienes (LTs), thromboxanes (TXs), and lipoxins (LXs) (Figure 1) (17). These AA-derived lipid meditators have both pro- and anti-inflammatory effects in the intestine.

AA is converted into LTB_4_ by LOX activity. The LTB_4_-BLT1 axis plays a key role in the development of inflammatory diseases including inflammatory bowel disease by stimulating the recruitment of inflammatory cells and the production of pro-inflammatory cytokines (18–20). LTB_4_ also activates another receptor BLT2 which is a high affinity receptor for 12-hydroxy-heptadecatrienoic acid (12-HHT). In contrast to pro-inflammatory role of BLT1, BLT2-deficient mice show transepidermal water loss, suggesting its anti-inflammatory role in the skin (21). Indeed, BLT2-mediated pathway induced the expression of claudin-4 for enhancement of epithelial barrier (21).

AA is converted into PGs by COX activity, which generate PGD_2_ and PGE_2_ as the representative lipid mediators. The PGD_2_-chemoattractant receptor-homologous molecule expressed on Th2 cells (CRTH2) pathway induces dextran sodium sulfate (DSS)- and trinitrobenzene sulfonic acid (TNBS)-induced colitis (22, 23). Eosinophil infiltration into colon is inhibited by CRTH2 antagonist treatment in TNBS-induced colitis (23). In contrast to pro-inflammatory properties, the PGD_2_-DP axis reduces granulocyte infiltration into the colonic mucosa in the mouse model of TNBS-induced colitis and colitis-associated colorectal cancer (24, 25) These opposing roles of CRTH2 and DP in chemotaxis are explained by different usage of G proteins. CRTH2 is coupled with Gα_i_ while DP is coupled with Gα_s_, which induces decreased and increased in cAMP levels, respectively (26). Consistent with these findings when PGD_2_ acted on neutrophils CRTH2 pathway, it induced neutrophil migration to the intestinal lamina propria in the DSS-induced colitis model (22).

PGE_2_ stimulates four distinct types of receptors EP1 to EP4. The PGE_2_-EP2 axis in neutrophils and tumor-associated fibroblasts promotes colon tumorigenesis by inducing expression of inflammation- and growth-related genes, including tumor necrosis factor (TNF)-α, interleukin (IL)-6, and Wnt5A (27). In contrast to EP2-mediated carcinogenic effects, EP3-mediated signals show anti-carcinogenic effects, which are consistent with different types of G protein pathways; EP2 activates Gα_s_, while EP3 activates Gα_i_ (27).

Therefore, it is suggested that the opposing roles in pro- and anti-inflammation of ω6-PUFAs derived lipid mediators are determined by target cell types and receptor types.

In addition to these factors, cellular source of PGD_2_ affects in its activity in pro- and anti-inflammation in croton oil-induced skin inflammation model (28). In the initial phase of the dermatitis when few inflammatory cells exist in the skin, endothelial cells show highest COX-2 activity and produce PGD_2_, which leads to DP activation on endothelial cells, and inhibits vascular leakage. On the other hand, in the late phase of the dermatitis, many types of hematopoietic inflammatory cells produce PGD_2_, which stimulate CRTH2 on inflammatory cells for infiltration to the inflamed skin, and exacerbates skin inflammation (28, 29). These findings suggest that stage of inflammatory process is a determinant of the effects of AA-derived metabolites through distinct site of the mediator production.

## Dietary ω3-PUFAs Inhibit the Development of Allergic Disease

We and others have shown the anti-inflammatory and anti-allergic effects of dietary ω3-PUFAs (4, 7, 8, 12, 13, 30–34).

Fish oil is a representative ω3-PUFA-rich dietary oil which contains plenty amount of EPA and DHA. Dietary fish oil ameliorated asthma by decreasing eosinophil infiltration, mucus production, and peribronchiolar fibrosis, which was associated with inhibition of cytokine production by downregulation of nuclear factor (NF)-κB and GATA-3 (30). These anti-allergic effects may be caused by decreased amount of ω6-PUFA-derived lipid mediators such as PGD_2_, LTB_4_, and LTE_4_ which exacerbate airway inflammation and increasing ω3-PUFA-derived lipid mediators, for example, RvD1 is reported to decrease allergic airway responses (6, 35, 36). Further, fish oil-fed mice reduced acute allergic skin response in food allergy model sensitized by peanut and whey by reducing mucosal mast cell protease-1 and antigen specific IgE in serum (31).

Linseed oil contains large amount of α-linolenic acid, which is converted into EPA and DHA in the body. One study reported that linseed oil-fed mice alleviated pollen-induced allergic conjunctivitis by decreasing the production of ω6-PUFA-derived pro-inflammatory lipid mediators, and reducing eosinophil infiltration into the conjunctiva (13). We also found that linseed oil-fed mice reduced allergic diarrhea in ovalbumin (OVA)-induced food allergy model (7). In this model, allergic diarrhea occurs as a consequence of a dominant Th2-type environment and the presence of allergen-specific serum IgE, which induces mast cell degranulation in the gut. We found that in linseed oil-fed mice, the Th1–Th2 balance, allergen-specific IgE level, and mast cell numbers in the gut did not change compared with those in soybean oil-fed mice in the OVA-induced food allergy model. However, we found that mast cell degranulation was profoundly inhibited in linseed oil-fed mice (7).

We also assessed fatty acid composition in intestinal tissues and found that the amounts of α-linolenic acid and its metabolites of EPA and DHA were increased in linseed oil-fed mice when compared with those in soybean oil-fed mice (7). In contrast, linoleic acid and AA levels were higher in soybean oil-fed mice than linseed oil-fed mice (7). Imaging MS analysis revealed that increased amounts of α-linolenic acid, EPA and DHA were found in the lamina propria compartment where large numbers of immune cells such as T cells, plasma cells, and dendritic cells are present (7). These findings collectively demonstrated that the composition of essential fatty acids in dietary oils directly reflect the lipid composition in the gut, which, in turn, may influence the host immune system.

## ω3 Fatty Acid Metabolites Have Roles in Anti-Inflammation and Pro-Resolution

EPA and DHA are representative ω3-PUFAs, which compete with AA in the AA cascade. Therefore, it has long been considered that the beneficial effects of dietary ω3-PUFAs against inflammatory diseases stem from decreased amounts of AA-derived eicosanoids. In addition, recent technology developments in LC and MS have led to the identification of trace and novel lipid mediators, including Rvs, PDs, and MaRs, which are produced from EPA and DHA in the body (37). These metabolites have anti-inflammatory or pro-resolution properties (or both) and are known as SPMs (Figure 1) (37). Although the receptors for SPMs have not been fully elucidated, some SPMs have been shown to interact with specific receptors. For example, Rvs derived from EPA and DHA use distinct types of receptors. RvE1 interacts with BLT1 and ChemR23, while RvD1 interacts with G-protein-coupled receptor (GPR) 32 and ALX (38, 39).

Examples of how SPMs affect intestinal inflammation include their involvement in the RvE1–ChemR23 axis, which actively inhibits colonic inflammation in the DSS-induced colitis model by suppressing the TNF-α-induced nuclear translocation of NF-κB and the expression of inflammatory cytokines, including TNF-α and IL-12p40, from macrophages (40). Furthermore, RvE1 and PD1 enhance the resolution of inflammation by stimulating macrophage phagocytosis of apoptotic cells in zymosan-induced peritonitis (41, 42). MaR1 is reported to attenuate both DSS- and TNBS-induced colitis by inhibiting NF-κB activation and inflammatory cytokine production (43). Thus, multiple types of SPMs exert their anti-inflammatory properties by using different mechanisms for the regulation of colitis.

## 17,18-Epoxyeicosatetraenoic Acid is a New Class of Anti-Allergy Lipid Mediator

**Figure 2:**
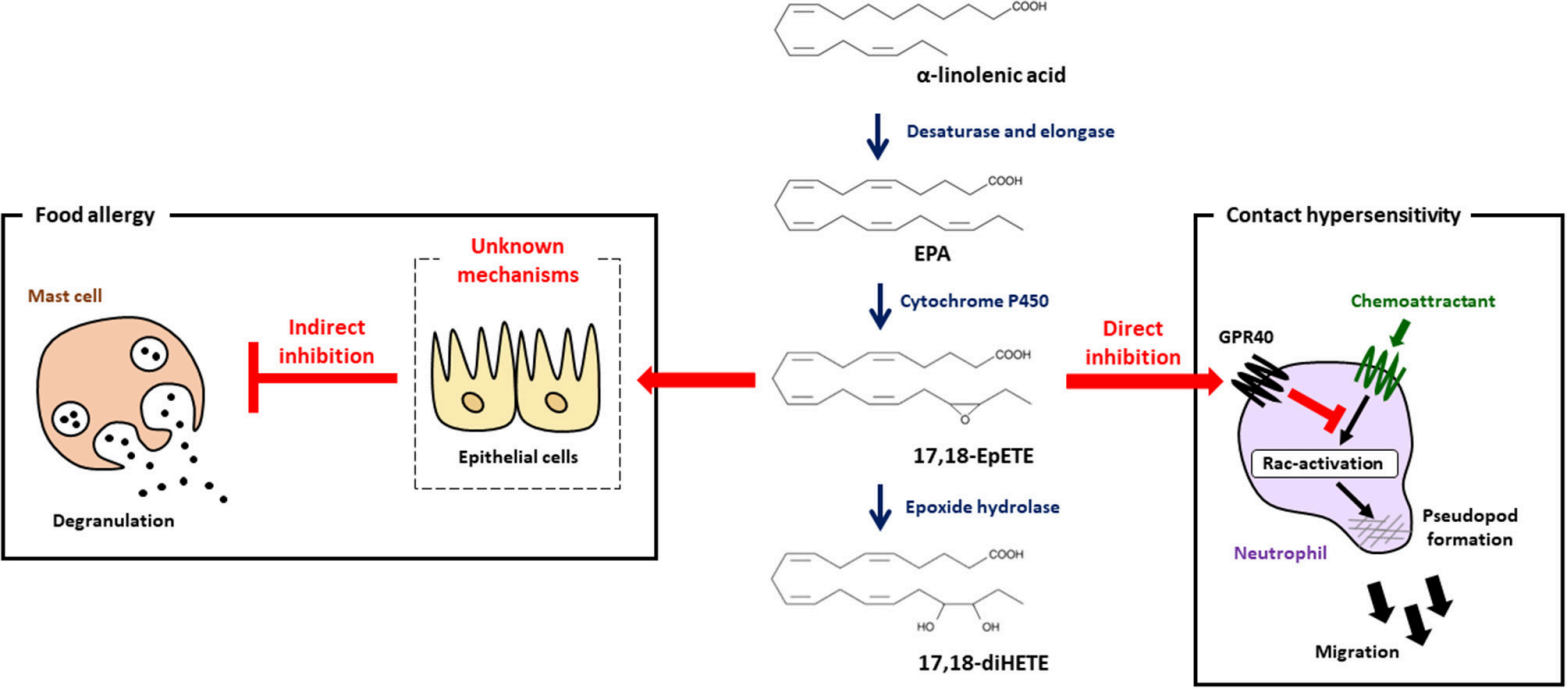
17,18-EpETE is a new class of anti-allergy and anti-inflammatory lipid mediator. 17,18-EpETE is produced by CYP from EPA. 17,18-EpETE suppresses contact hypersensitivity by reducing neutrophil infiltration into the skin by inhibiting Rac activation and migration through GPR40 signaling. 17,18-EpETE also indirectly inhibits the development of food allergy by inhibiting mast cell degranulation. Given that mast cells do not express GPR40, the detailed mechanisms responsible for this inhibition of mast cell degranulation remain unclear.

As mentioned above, dietary linseed oil inhibited the development of food allergy with increased amounts of α-linolenic acid, EPA and DHA in the intestine (7), which prompted us to investigate mediator profiles by using LC-MS/MS analysis. We found that 17,18-EpETE was the metabolite whose levels increased the most in the gut of linseed oil-fed mice (7). When 17,18-EpETE was intraperitoneally injected into soybean oil-fed mice, development of allergic diarrhea and degranulation of mast cells were inhibited, which was similar to observation in linseed oil-fed mice (Figure 2) (7). Consistent with its action at the late stage of the allergic response, 17,18-EpETE was effective as a prophylactic and a therapeutic treatment for food allergy (7).

## 17,18-EpETE Ameliorates Contact Hypersensitivity Through GPR40-Mediated Inhibition of Neutrophil Migration

To evaluate the biological role of 17,18-EpETE in the regulation of other types of allergic inflammatory disease, we examined the effect of 17,18-EpETE on the regulation of contact hypersensitivity (CHS) in the hapten-induced CHS model. We found that 17,18-EpETE showed both prophylactic and therapeutic anti-inflammatory effects on CHS in mice and cynomolgus macaques (44). 17,18-EpETE did not affect T cell or dendritic cell functions, including inducible skin-associated lymphoid tissue formation, but it did selectively inhibit neutrophil infiltration into the skin (44). Indeed, 17,18-EpETE reduced neutrophil mobility by inhibiting Rac-activation and pseudopod formation in a GPR40-dependent fashion (44). Consistent with this selective influence on neutrophils, GPR40 was highly expressed by neutrophils, but not T cells or other leukocytes in the skin. It is worth noting that mast cells do not express GPR40; so, given that mast cell degranulation was inhibited by 17,18-EpETE treatment in the food allergy model (7, 44), this finding suggests that 17,18-EpETE inhibits mast cell degranulation indirectly (Figure 2). Of note, the activation of GPR40 in intestinal epithelial cells has been reported to improve intestinal barrier function by enhancing occludin expression (45). Therefore, it is likely that the improvement in intestinal barrier function induced by 17,18-EpETE via GPR40 in epithelial cells led to decreased allergen penetration, which, in turn, resulted in decreased mast cell degranulation and inhibited food allergy development.

## Structure-Activity Relationships Among the GPR40-Dependent Anti-Allergic and Anti-Inflammation Effects of 17,18-EpETE

17,18-EpETE is further metabolized by soluble epoxide hydrolase to 17,18-dihydroxy-eicosatetraenoic acid (17,18-diHETE). However, 17,18-diHETE has little effect on the development of food allergy, and 14,15-epoxyeicosatetraenoic acid (14,15-EpETE), which has an epoxy structure at the ω6 position, also lacks the ability to inhibit food allergy (7). In addition, 17,18-diHETE has little effect on the development of CHS (44). Although 17,18-EpETE activates GPR40, 17,18-diHETE does not activate GPR40, which is consistent with its lack of anti-allergic and anti-inflammatory properties (7, 44). These findings therefore suggest that the 17,18-epoxy ring structure at the ω3 position in EPA is important for GPR40-mediated anti-allergic and anti-inflammatory activity.

17,18-EpETE is synthesized from EPA through the enzymatic activity of CYP and has two isomers, 17(*S*),18(*R*)-EpETE and 17(*R*),18(*S*)-EpETE. Among the CYP subfamilies in mice, five CYP isoforms (Cyp1a2, 2c50, 4a12a, 4a12b, and 4f18) are known to convert EPA into 17,18-EpETE (46). Cyp1a2 displays high stereoselectivity for producing 17(*R*),18(*S*)-EpETE, whereas Cyp4f18 displays stereoselectivity for producing 17(*S*),18(*R*)-EpETE (46). In contrast, Cyp2c50, Cyp4a12a, and Cyp4a12b display less stereoselectivity and produce a mixture of 17(*S*),18(*R*)-EpETE and 17(*R*),18(*S*)-EpETE (46). 17(*R*),18(*S*)-EpETE, but not 17(*S*),18(*R*)-EpETE, is a potent vasodilator (47). Indeed, 17(*R*),18(*S*)-EpETE activates calcium-activated potassium channels, which lead to relaxation of rat cerebral artery vascular smooth muscle cells (47). Whether stereoselectivity of 17,18-EpETE contributes to the anti-allergy and anti-inflammatory effects of 17,18-EpETE have not been evaluated in food allergy and CHS, because we used racemic compounds in our studies (7, 44). The CYP isoform and polymorphisms determine the metabolic properties of CYP and stereoselectivity. Therefore, the anti-allergic and anti-inflammatory health benefits derived from ω3-PUFA intake may be influenced by the expression levels of the various types of CYP in the body.

CYP is also found in microorganisms. For example, it has been reported that bacterial CYP (e.g., BM-3 derived from *Bacillus megateirum*) metabolizes PUFAs and produces hydroxy and epoxy fatty acids (48). *Bacillus, Streptomyces, Pseudomonas*, and *Mycobacterium* also have CYP (49–53). These findings suggest that many types of microorganisms are involved in lipid metabolism. In addition, other metabolic enzymes, such as COX and LOX, are thought to be expressed by some bacteria, including *Pseudomonas aeruginosa, Shewanella woodyi, Mytococcus fulrus*, and *Burkholderia thailandensis* (54, 55). Some microorganisms described above are present in environment, suggesting that in addition to mammalian expression of metabolic enzymes, various microorganisms may be a determinant of the efficacy of ω3-PUFA in the context of the regulation of inflammation.

## Bacterial-Conjugated Linoleic Acid has a Role in Anti-Inflammation

**Figure 3:**
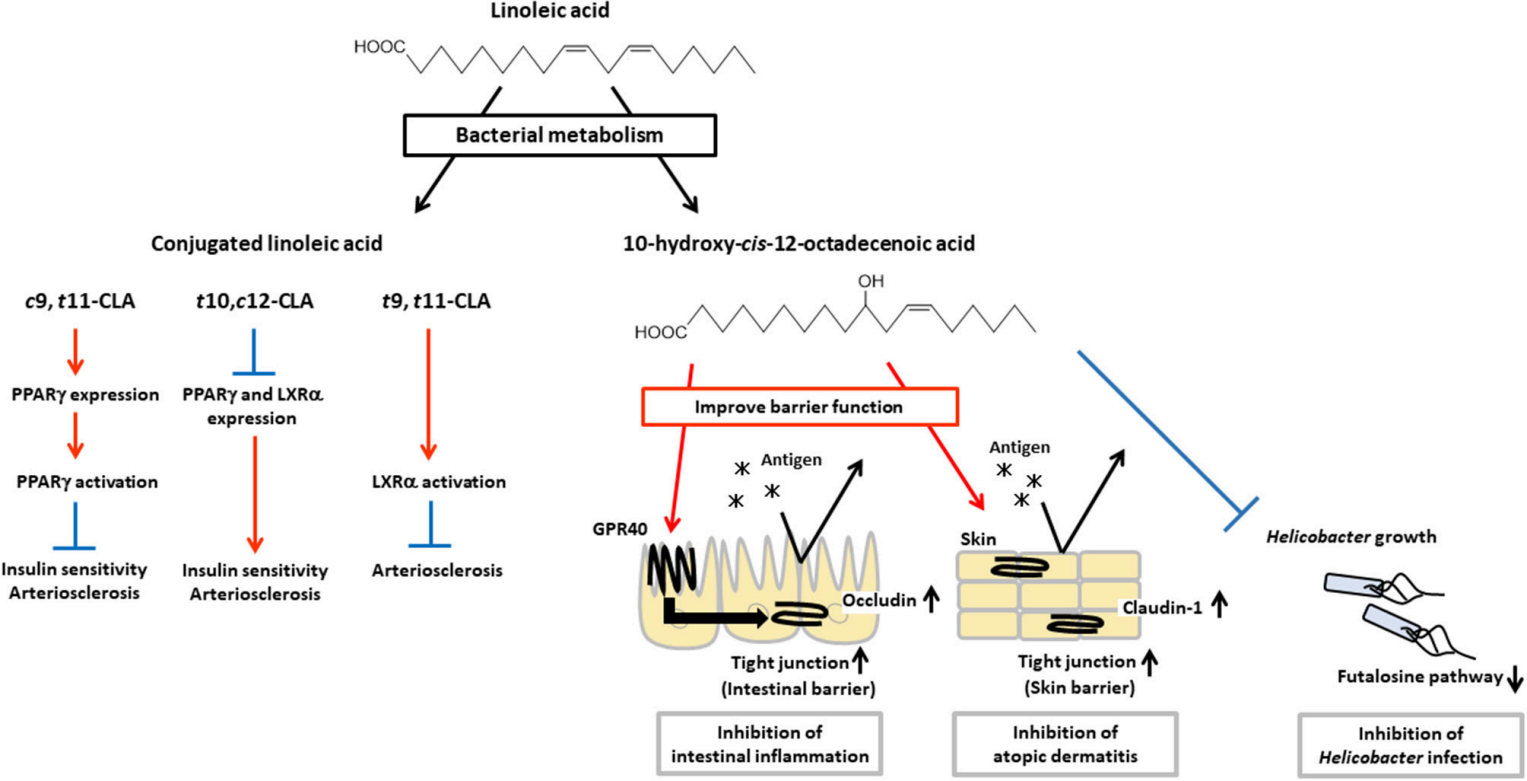
Physiological functions of CLA and HYA. CLA and HYA are produced from linoleic acid by intestinal bacteria. *c*9,*t*11-CLA ameliorates insulin sensitivity and prevents atherosclerosis, *t*10,*c*12-CLA deteriorates insulin sensitivity and promotes atherosclerosis, and *t*9,*t*11-CLA prevents atherosclerosis. HYA enhances intestinal barrier function by increasing occludin expression and inhibiting intestinal inflammation in a GPR40-dependent manner. HYA inhibits atopic dermatitis by increasing claudin-1 expression and enhancing skin barrier function. HYA also inhibits gastric *Helicobacter* infections by blocking the bacterial futalosine pathways.

Intestinal bacteria have been shown to express unique unsaturated fatty acid-metabolic enzymes and to produce bioactive lipid mediators that are not generated by mammalian cells (Figure 3). Ruminal bacteria including *Butyrivibrio, Lactobacillus*, and *Megasphaera* can produce conjugated linoleic acid (CLA), which is an isomer of linoleic acid that has conjugated double bounds (56–58). It is known that CLA has some isomers such as *cis*-9-*trans*-11-octadecenoic acid (*c*9,*t*11-CLA), *trans*-10-*cis*-12-octadecenoic acid (*t*10,c12-CLA) and *trans*-9-*trans*-11-octadecenoic acid (*t*9,*t*11-CLA). These isomers have different activities for insulin sensitivity and atherosclerosis.

For example, *c*9,*t*11-CLA shows beneficial effects on insulin sensitivity by enhancing glucose uptake and adipokine production such as leptin and adiponectin, and on atherosclerosis by suppressing macrophage infiltration and activation, and reducing plaque development through an increase in expression of PPARγ, while *t*10,c12-CLA shows adverse effects through a decrease in expression of PPARγ (59–63). In addition, *t*10,c12-CLA reduces expression of liver X receptor α (LXRα) which induces expression of ATP-binding cassette (ABC) transporter A1, ABCG1, and sterol regulatory element binding protein 1c which involved in reverse cholesterol transport (64, 65). Therefore, *t*10,c12-CLA shows pro-atherosclerosis effects (66–68). On the other hand, *t*9,*t*11-CLA is effective for the treatment of atherosclerosis by activation of LXRα (69). These results indicate that each isomers exert different bioactivities through distinct transcriptional regulation and activation of PPARγ and LXRα for the control of insulin sensitivity and atherosclerosis.

Compared with chemical production, microbial fermentation offers better ways to produce isomer-specific CLAs. The CLA isomers are produced at different ratios, depending on the type of bacteria. *Lactobacillus* strains (*L. acidophilus, L. plantarum, L. casei, L. reuteri, L. rhamnosus*, and *L. pentosus*), *Bifidobacterium* strains (*B. dentium, B. breve*, and *B. lactis*), and *Propionibacterium freudenreichii* can convert linoleic acid to *c*9,*t*11-CLA and *t*10,c12-CLA, and these bacteria produce higher levels of *c*9,*t*11-CLA than of *t*10,c12-CLA (15, 57, 70–72). Some *Lactobacillus* and *Bifidobacterium* strains also produce *t*9,*t*11-CLA with *c*9,*t*11-CLA and/or *t*10,c12-CLA (57). *L. paracasei* and *B. bifidum* produce *c*9,*t*11-CLA stereoselectively, whereas *Megasphaera eldsenii* produces *t*10,c12-CLA stereoselectively (71, 73). Given that these CLAs have different biological activities which depend on their 3D-structure, it is important to select appropriate bacteria as a probiotics or producer for obtaining required beneficial effects.

## Bacterial Production of Unique Hydroxy and Oxo Fatty Acids and Their Multiple Biological Activities

*L. plantarum*, an intestinal bacteria, produces hydroxy fatty acids (i.e., 10-hydroxy-*cis*-12-octadecenoic acid [HYA], 10-hydroxy-*trans*-11-octadecenoic acid [HYC], 10-hydroxy-octadecanoic acid [HYB]) and oxo fatty acids (10-oxo-*cis*-12-octadecenoic acid [KetoA], 10-oxo-*trans*-11-octadecenoic acid [KetoC], 10-oxo-octadecanoic acid [KetoB]) as intermediate products of CLA production (16). Recently, these metabolic intermediates have been shown to contribute to the regulation of host health and diseases. HYA is the first metabolite produced from linoleic acid by *L. plantarum*, and it enhances intestinal barrier function and suppresses the development of DSS-induced colitis in mice in a GPR40-dependent manner (45). Furthermore, HYA prevents *Helicobacter* infections by blocking their futalosine pathways, which is an alternative menaquinone biosynthetic pathway and an essential metabolic pathway for the growth of *Helicobacter*. Moreover, HYA treatment suppresses the formation of lymphoid follicles in the gastric mucus layer after *H. suis* infection, and therefore HYA treatment protects mice against the formation of gastric mucosa-associated lymphoid tissue lymphoma induced by infection with *Helicobacter* (74). HYA also ameliorates the pathological scores of atopic dermatitis in NC/Nga mice by decreasing plasma IgE levels and reducing mast cell infiltration into the skin (75, 76). KetoA enhances adiponectin production and glucose uptake in a proliferator-activated receptor γ (PPARγ)-dependent manner, and is effective for the prevention and amelioration of metabolic abnormalities associated with obesity (77).

The production of these hydroxy and oxo fatty acids depends on the unique bacterial enzymes CLA-HY (unsaturated fatty acid hydratase), CLA-DH (hydroxy fatty acid dehydrogenase), CLA-DC (isomerase), and CLA-ER (enone reductase) in *L. plantarum* AKU1009a (16, 78). The hydroxy activity is found not only in *Lactobacillus* but also in a broad spectrum of bacteria. Oleate hydratase belongs to the FAD-dependent myosin cross-reactive antigen (MCRA) protein family, which is found in gram-positive and -negative bacteria; it catalyzes the conversion of linoleic acid to HYA. For example, *Lactobacillus, Bifidobacterium, Streptococcus*, and *Stenotrophomonas* bacteria are reported to have MCRA, and indeed they have the ability to produce HYA (79–82).

Together, these findings indicate that intestinal bacteria metabolize dietary lipids and produce lipid metabolites that can regulate host immune systems. Therefore, to obtain beneficial lipid metabolites and regulate intestinal inflammation, we need to consider not only host enzymes but also enzymes produced by intestinal bacteria. In addition, we must consider how dietary lipid intake causes changes in the intestinal microbiota.

## Conclusion

Recent technological developments in lipidomics research initiated a new era of lipid biology by helping researchers to identify novel lipid metabolites from ω3- and ω6-PUFAs, which actively regulate the host immune system and play important roles in the control of health and diseases. Given that the production of lipid metabolites is influenced by complex factors, including diet, intestinal bacteria, and enzyme expression, combined studies on nutrition, metabolomics, and the metagenomics of the microbiota, as well as informatics, may provide powerful insights to further our understanding of the lipid network in the host immune system.

## Author Contributions

All authors listed have made a substantial, direct and intellectual contribution to the work, and approved it for publication.

### Conflict of Interest Statement

The authors declare that the research was conducted in the absence of any commercial or financial relationships that could be construed as a potential conflict of interest.

## Abbreviations

12-HHT: 12-hydroxy-heptadecatrienoic acid
14,15-EpETE: 14,15-epoxyeicosatetraenoic acid
17,18-EpETE: 17,18-epoxyeicosatetraenoic acid
17,18-diHETE: 17,18-dihydroxy-eicosatetraenoic acid
AA: arachidonic acid
CHS: contact hypersensitivity
CLA: conjugated linoleic acid
COX: cyclooxygenase
CRTH2: chemoattractant receptor-homologous molecule expressed on Th2 cells
CYP: cytochrome P450
DC: dendritic cell
DHA: docosahexaenoic acid
DSS: dextran sodium sulfate
EPA: eicosapentaenoic acid
GPR: G-protein-coupled receptor
HYA: 10-hydroxy-*cis*-12-octadecenoic acid
HYB: 10-hydroxy-octadecanoic acid
HYC: 10-hydroxy-*trans*-11-octadecenoic acid
IL: interleukin
KetoA: 10-oxo-*cis*-12-octadecenoic acid
KetoB: 10-oxo-octadecanoic acid
KetoC: 10-oxo-*trans*-11-octadecenoic acid
LC: liquid chromatography
LOX: lipoxygenase
LT: leukotriene
MaR: maresin
MCRA: myosin cross-reactive antigen
MS: mass spectrometry
NF: nuclear factor
OVA: ovalbumin
PD: protectin
PG: prostaglandin
PPAR: peroxisome proliferator-activated receptor
PUFA: polyunsaturated fatty acid
Rv: resolvin
SPM: specialized pro-resolving lipid mediator
TNF: tumor necrosis factor
TX: thromboxane.
